# Climate change adaptation for livestock production in southern Australia: transdisciplinary approaches for integrated solutions

**DOI:** 10.1093/af/vfab046

**Published:** 2021-10-20

**Authors:** Brendan R Cullen, Margaret Ayre, Nikki Reichelt, Ruth A Nettle, Gillian Hayman, Daniel P Armstrong, Ruth Beilin, Matthew T Harrison

**Affiliations:** 1 Faculty of Veterinary & Agricultural Sciences, The University of Melbourne, Parkville, Victoria, Australia; 2 Gillian Hayman, Facilitation & Project Services, Fish Creek, Victoria, Australia; 3 D-ARM Consulting, Jindivick, Victoria, Australia; 4 School of Ecosystem and Forest Sciences, Faculty of Science, The University of Melbourne, Parkville, Victoria, Australia; 5 Tasmanian Institute of Agriculture, University of Tasmania, Burnie, Tasmania, Australia

**Keywords:** climate extremes, dairy, grazing, multidisciplinary, pasture

**Implications**

- Climate change will affect the seasonal production of pastures for livestock feed, and adaptation is required. Adaptation will require changes to production systems and new skills in the workforce.
- Transdisciplinary research approaches are needed to address the adaptation challenge. These approaches combine farmer knowledge with farm systems and economic analysis to assess the impacts of a changing climate and potential adaptation benefits, and social research to address the nontechnical drivers of change.
- Research is required to integrate knowledge from different disciplines. A case study of adaptation research in the Australian dairy industry is used to highlight the transdisciplinary approach.

## Introduction 

Grazing lands (savannas, grasslands, prairies, steppe, and shrublands) cover 45% of the earth’s land surface (excluding Antarctica) (Reid et al., 2008) and are important sources of feed for livestock production which are estimated to supply 17% of global human energy requirement and support livelihoods in both developed and developing countries (Herrero et al., 2009). Grazing-based livestock production systems utilize native grasslands and sown pastures as a primary feed source for animals. These systems span both a diverse climatic range from cool temperate to tropical and production environments from subsistence farming to large, intensively grazed systems. Grasslands are reliant on the climate, primarily rainfall and temperature, to produce feed for livestock, and climate variability has a large impact on the production and profitability of such systems (e.g., Chapman et al., 2009). Projected climatic changes globally, including warmer temperatures, increasing atmospheric carbon dioxide concentrations, and changes in seasonal rainfall patterns, will impact the seasonal pasture growth and livestock production. While a warmer climate will also directly impact animals, such as through increased periods of heat stress (e.g., Chang-Fung-Martel et al., 2017), the focus of this article is on grazing-based production systems rather than animal responses.

Adaptation to climate change is defined as “the process of adjustment to actual or expected climate and its effects” (IPCC, 2014). In livestock production systems, the changing climate may directly affect the pattern of pasture growth, livestock production, farm profitability, and environmental sustainability. Adaptation is required and may involve changes that require the development of new skills for people working in these industries. The challenge for adaptation in livestock businesses, and agricultural systems more broadly, goes beyond technical changes in inputs and outputs of the production system, to considering the implications of a changing climate for farm business profitability, the people working in these industries, and their communities. Addressing the multiple challenges of climate change adaption requires a transdisciplinary approach (Klenk and Meehan, 2015), yet there are few examples of how transdisciplinary research teams can work together to effectively address these challenges for livestock businesses. In this paper, the impacts of climate change on pasture and animal production are briefly reviewed and a case study of transdisciplinary climate change adaptation in south-eastern Australia is presented.

## Climate Change Impacts and Adaptation for Grazing Systems

**Figure 1.**
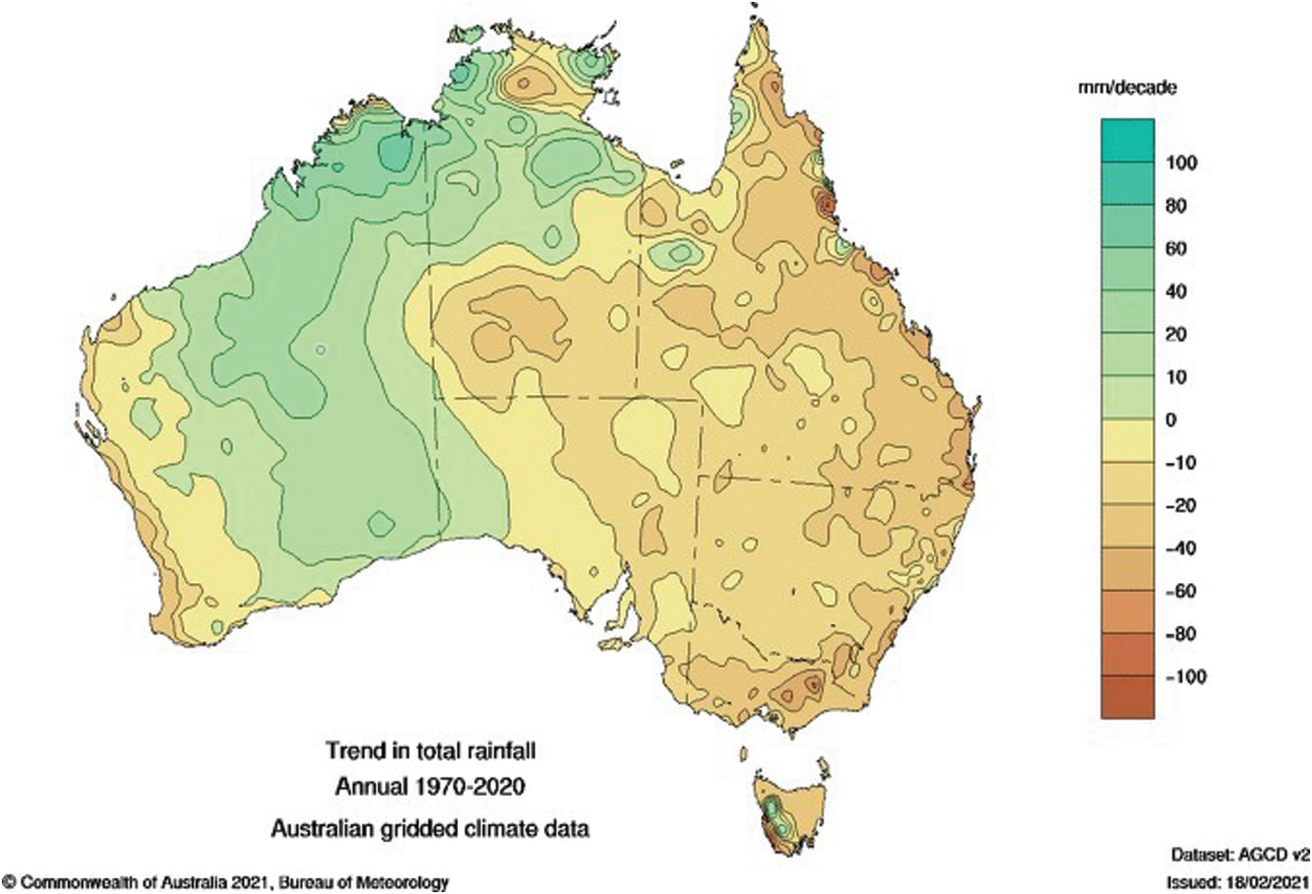
Trends in annual rainfall change (mm/decade) from 1970 to 2020 in Australia. Source: Bureau of Meteorology, Australia.

Climate change projections consistently indicate that global temperatures and atmospheric carbon dioxide concentration will increase; however, the projections for rainfall change are less clear and are likely to vary regionally (IPCC, 2014). In southern Australia, a decline in rainfall since the 1970s has been observed (Figure 1), and further declines in winter and spring rainfall are predicted. Coupled with gradual changes to the average climate are increases in the frequency and severity of extreme climate events, including heatwaves, drought, and intense rainfall. Changes in climate variability and extreme events are likely to have a greater impact on agricultural systems than changes in climate averages (Thornton et al., 2014; Harrison et al., 2016).

The impact of climate change on grazing systems will depend not only on the amount that the climate changes but also on the characteristics of the existing climate and production systems that determine how resilient they are to change, and on the capacity of farmers to adapt. For example, tropical and subtropical pastures are tolerant of high temperatures so are unlikely to be negatively impacted by warming, but temperate pasture species such as perennial ryegrass (*Lolium perenne*) are susceptible (e.g., Langworthy et al., 2018). Forage production and quality of temperate pastures are also likely to decline due to a contraction of the growing season (Cullen et al., 2009; Moore and Ghahramani, 2013), and changes in species composition may occur.

In regions with increasing temperatures and declining rainfall, such as southern-eastern Australia, the impacts of climate change on pasture production and livestock are already being observed. A shift in the seasonal distribution of pasture production (more growth in winter but a contraction of the growing season) with increasing year-to-year variability in pasture production has been modeled (Perera et al., 2020). Shorter growing seasons also create a risk of pastures providing low levels of ground cover, thus exposing the soil to degradation through erosion unless stocking rates of livestock are reduced (Moore and Ghahramani, 2013). A range of potential adaptation options have been identified to reduce the impact of the changing climate in the region, including increasing soil fertility, use of summer active pasture species, and animal genetic improvement, but these options become less effective with larger changes to the climate (Ghahramani and Moore, 2015). The impacts of climate change are often specific to local conditions, and the development of adaptation options also needs to be done in a way that is both context and application specific.

A key role of climate change adaptation research in agriculture internationally has been to support the adaptive capacity of systems. Adaptive capacity has been defined as the “resources available for adaptation to climate change and variability or other related stresses, as well as the ability of a system to use these resources effectively in the pursuit of adaptation” (Brooks and Adger, 2005). Adaptations to climate change can include incremental, systemic, and transformative changes to farm systems, the latter describing higher-risk, long-term changes to farm systems (Vermeulen et al., 2018). Previous work has shown that there are many incremental options for adaptation in agricultural systems that will have substantial benefits under moderate climate change (up to 2 °C warming), but their efficacy under higher warming scenarios is limited (Howden et al., 2007; Ghahramani and Moore, 2015). In the latter cases, more transformational changes in resource allocation need to be considered, including changing location to more favorable climatic regions, diversification of production systems (e.g., changing mix of enterprises), and livelihoods (Howden et al., 2007).

Adaptive capacity can be supported through transdisciplinary collaboration in research that combines the knowledge and skills of scientific and professional practice experts (such as farmers, advisors, and policy makers) in the development of scientifically robust and practical options for farming systems change and improved management (Rickards et al., 2014). Transdisciplinary research aims to integrate different academic and expert practitioner knowledge/s in a process of collaboration between scientists and nonscientists on a specific real-world problem (Walter et al., 2007) that is refined iteratively over several cycles between researchers and end users. The process of transdisciplinary collaborative research must be carefully designed to ensure that contributions of participants from outside the academic disciplines are integrated because this has a strong bearing on the quality and feasibility of adaptation pathways identified (Meinke et al., 2009; Ayre and Nettle, 2015).

In the following section, we illustrate the transdisciplinary process using a case study conducted in the Australian dairy industry. The “Dairy Businesses for Future Climates” (DBFC) project integrated quantitative biophysical and economic modeling of dairy farming systems with qualitative social science and expert dairy farmer, farm advisor, and dairy industry professional knowledge, to determine a set of actionable, realistic, and industry-validated climate change adaptation scenarios (“development options”) for dairy businesses.

## Transdisciplinary Approaches to Climate Change Adaptation Research—A Case Study of the “DBFC” Project

### Project background and design

**Figure 2.**
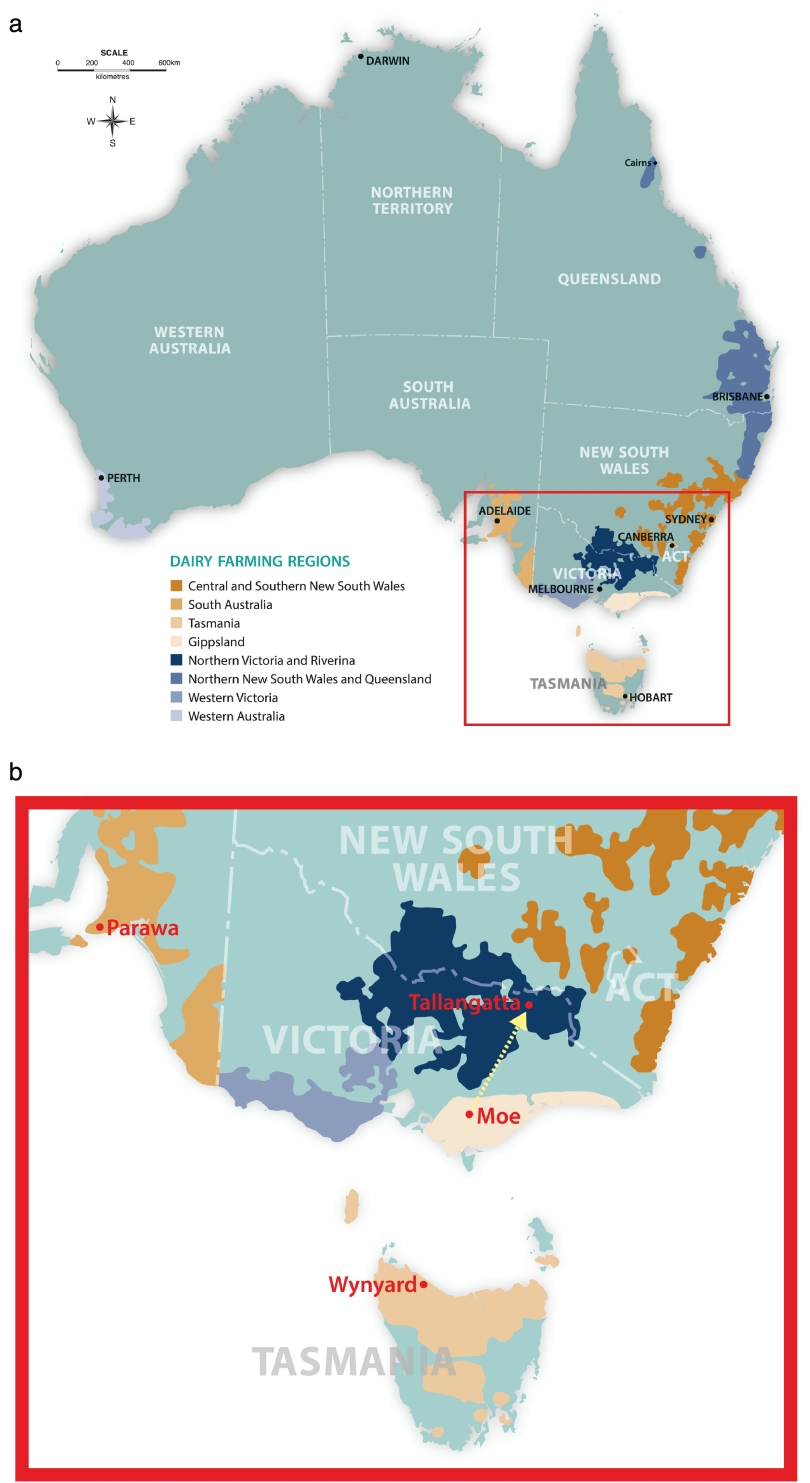
Maps of (a) Australian dairy farming regions and (b) the location of the three DBFC project case study farms. The dotted line in (b) indicates the Tallangatta site that was predicted to have a similar climate to Gippsland in 2040 and which is the focus of the “A trip to the future” section.

The Australian dairy industry is predominantly located in the south-eastern states (Figure 2a) in regions with temperate climates where moderate–high annual average rainfall (700+ mm) supports pasture growth over relatively long growing seasons (7–10 mo of the year) or where rainfall can be supplemented with irrigation. The majority of dairy farms maintain a high reliance on pasture as a source of feed for cows; however, there has been an intensification trend toward increased levels of concentrate feeding to support higher milk production per cow and high stocking rates, with some farms adopting partial or total mixed ration feeding (Clark et al., 2013). This trend of intensification—with fewer and larger farms, and a focus on maximizing production per unit of input—is a characteristic of many dairy industries around the world (Clay et al., 2020). However, there is evidence that more intensified farms with higher stocking rates and milk production have higher surpluses of nutrients such as nitrogen that may lead to poorer environmental outcomes (Gourley et al., 2012) including eutrophication, loss of ground cover, and soil erosion, if the infrastructure and management practice are inadequate. There may also be impacts of intensification on animal welfare, labor, skills requirements, and rural communities (Clay et al., 2020).

**Figure 3.**
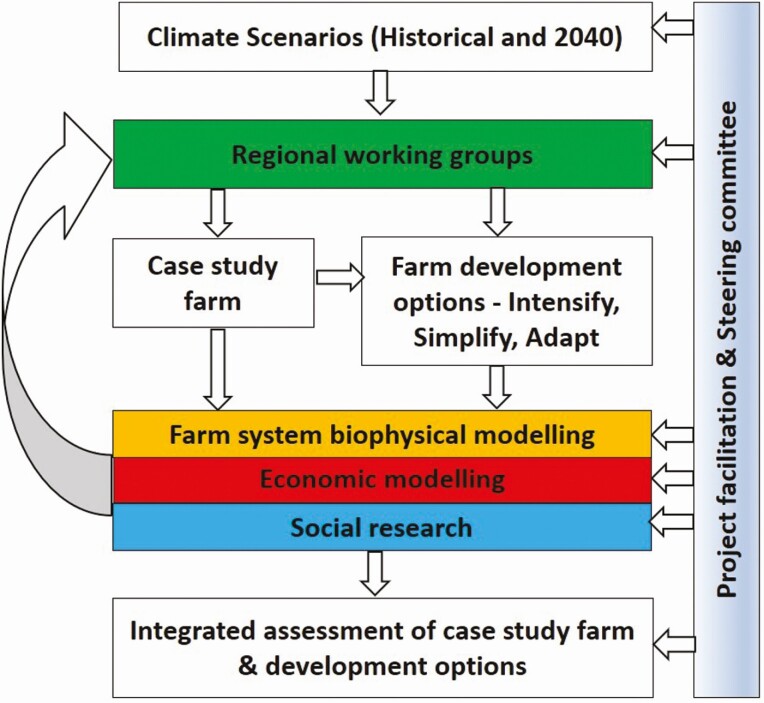
Schematic diagram of the transdisciplinary process used in the DBFC project. Different discipline groups are highlighted in different colors.

The DBFC project addressed the challenge of adaptation to a changing climate in south-eastern Australia by examining the interactions between dairy farming systems and a warmer, drier, and increasingly variable climate (Harrison et al., 2016). The project explicitly set out to consider the implications of dairy farm systems and climate changes on production, profit, and risk (measured by inter-annual variability of profit) from dairy farms as well as the implications for people, communities, and the environment. An outline of the structure of the project is shown in Figure 3, highlighting the transdisciplinary nature of the project and the key role that end users (dairy farmers) had in the regional working groups (Nettle et al., 2013). To cover the diversity of climate and farm systems, case study farms were investigated in the three of the major dairy regions: Moe in Gippsland, Victoria; Parawa on the Fleurieu Peninsula, South Australia; and Wynyard in north-west Tasmania (Figure 2b). The Gippsland and South Australian farms were rainfed production systems but the Tasmanian farm had irrigation.

**Figure 4.**
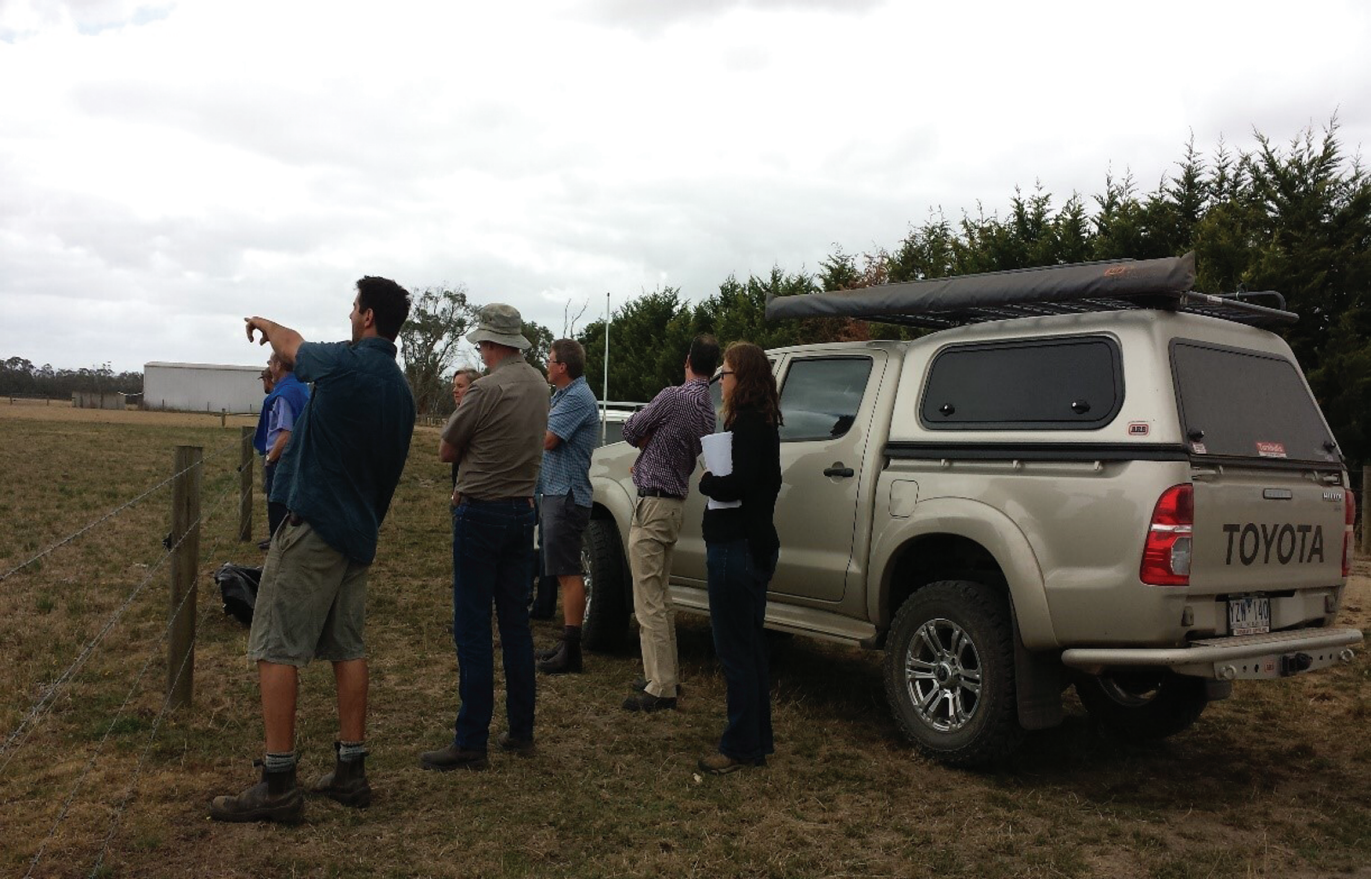
Gippsland regional working group members visited the case study farm to discuss the production system and identify potential adaptation options (Photo credit: G.H.).

**Figure 5.**
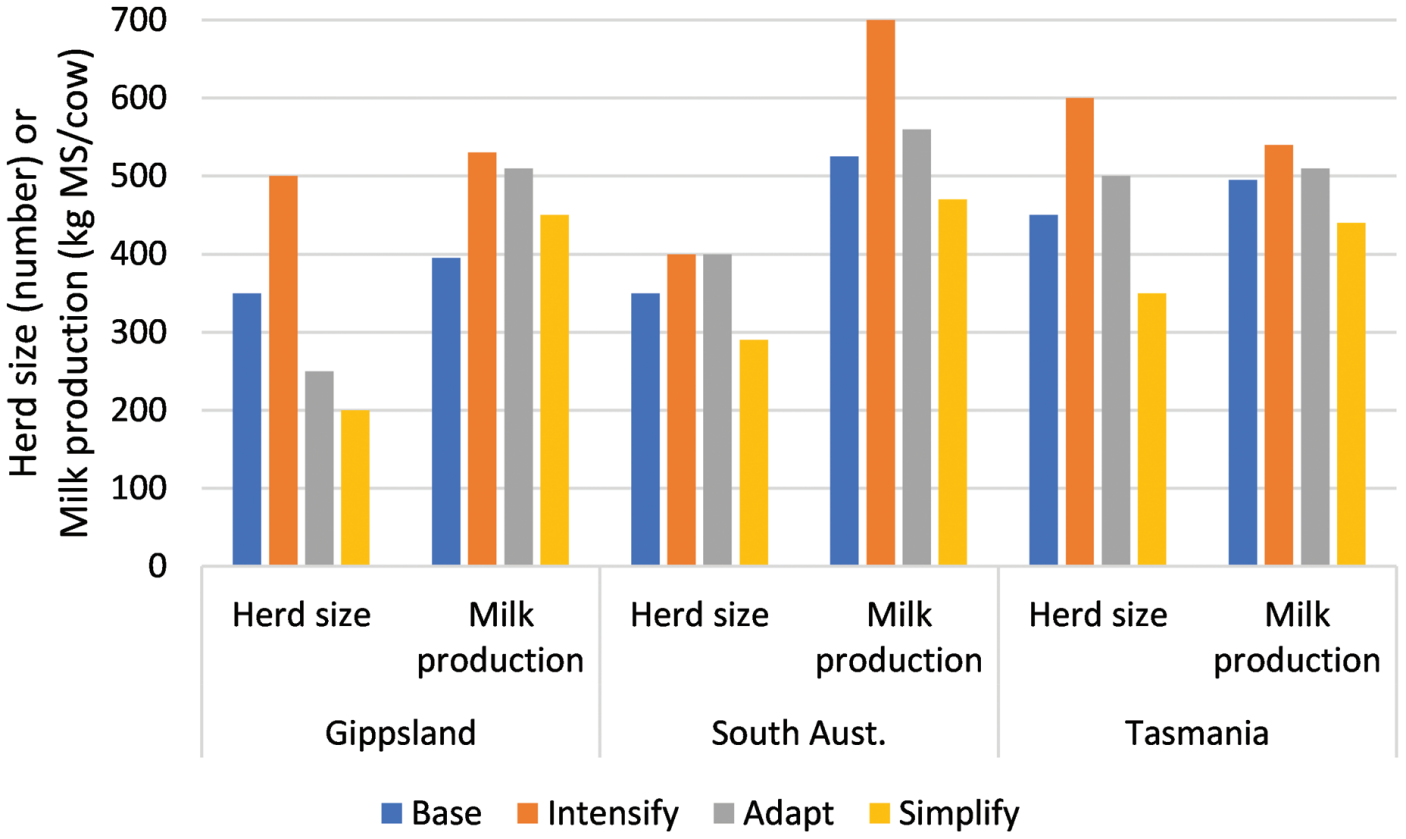
Herd size and annual milk production (kg milk solids [MS]/cow) of the Base (case study) farm and the Intensify, Adapt, and Simplify development options for the three DBFC regions. Further details are available in Harrison et al. (2017).

The key elements of the DBFC project design were:

- A transdisciplinary project team: including farmers, advisors, regional facilitators, and researchers from the disciplines of farm systems analysis, farm economics, and social science.
- Regional working groups: made up of local farmers and advisors who framed the adaptation problem in the context of local farm systems and end-user needs. These groups selected the case study farms and defined adaptation pathways (“farm development options”). The regional working groups met approximately six times each throughout the project (Figure 4).
- Real, local case study farms: a local farm that was representative of a well-managed dairy production system in each region. The case study (or “base”) farms were the basis for farm systems and economic analyses, social science analysis, and the modeling of “farm development options” as a response to predicted climate change impacts.
- Farm systems and economic modeling: this integrated modeling was used to predict impacts of the future climate on dairy production and profitability. This process utilized historical climate information and regional climate change projections for 2040 and 2080 to create future climate scenarios that captured both changes to the average seasonal climate and climate extremes (Harrison et al., 2016), then used biophysical modeling to estimate the impacts on farm production (including pasture consumption, supplementary feeding, and milk production) and economic analysis to incorporate risks associated with variability in milk prices and inputs costs (Harrison et al., 2017).
- Farm development options: these options explored three different adaptation pathways:
- ◦ “Intensification”—increased milk production from larger herds, increased use of concentrate feeding, and greater investment in machinery and infrastructure;
- ◦ “Adapt”—predicted changes in pasture growth rates were used to reorganize the resources on the farm (e.g., calving time) without substantial change in total milk production or investment in infrastructure; and
- ◦ “Simplify” (or extensification)—reduced herd size with greater reliance on “homegrown” feed, with no additional expenditure on infrastructure.
- A brief summary of key farm inputs (herd size and milk production) for the base farms and the development options for each region is shown in Figure 5. The options were defined differently in each region based on local industry practice. Farm systems and economic modeling was used to investigate the impact of climate change on the development options using the same approach outlined above.
- Cycles of feedback from the research teams to the regional working groups: iterative discussions were used to test and refine the assumptions used in the research. The farm systems and economic modeling results were used to have a more informed and meaningful discussion amongst the project team.
- Expert facilitation: to coordinate the inputs from the project team members, ensure clarity of communication, and facilitate network building and further learning opportunities for project participants (see example in text box).
- Inclusion of social science: that supported a participatory assessment of the farm development options by the regional working groups. This assessment explored the human and social capital dimensions of the options to understand the implications for farmers, their families, the regional community, and the wider dairy industry.

### Summary of project results

**Figure 6.**
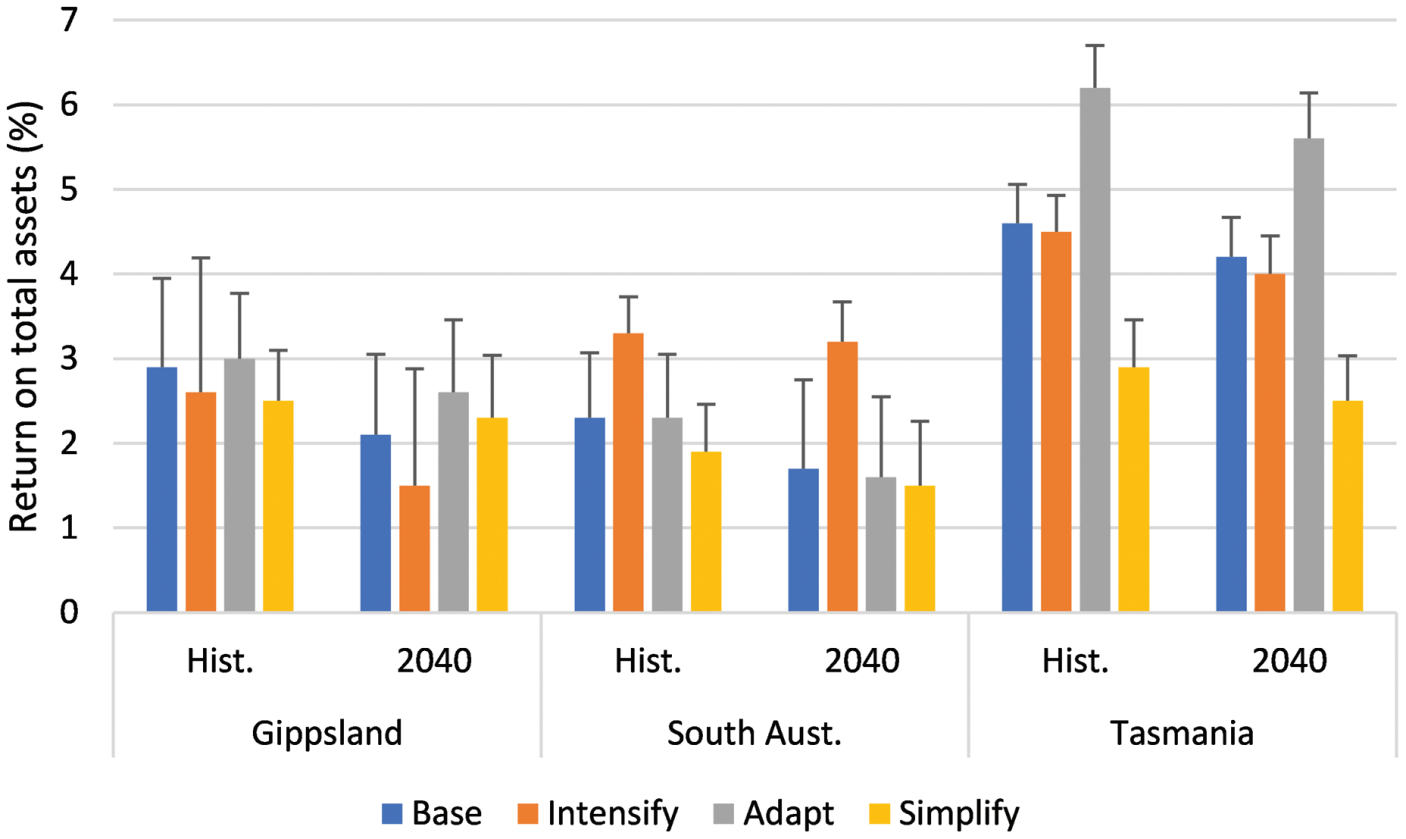
The annual average return on total assets (%) of the Base farm and Intensify, Adapt, and Simplify development options in each of the three regions under the historical and 2040 climate scenarios. The error bars show one standard deviation.

The results of the farm systems and economic modeling demonstrated that the warmer and drier climates predicted in 2040 would have a negative impact on pasture production and consumption at the two rainfed farms in Gippsland and South Australia leading to reduced and more variable profitability, but the impact was lower on the irrigated farm in Tasmania (Figure 6; Harrison et al., 2017). In general, Intensification options had the greatest inter-annual variability in profit and were the most affected by climate change, although the Intensified option in South Australia which was a total mixed ration system was an exception to this. The Adapt options showed some potential to reduce the climate change risk by better aligning the feed demand from cows with the changed pattern of pasture supply for example by switching from spring calving to autumn or implementing a split calving system (spring and autumn), whereas the Simplify options had the lowest and least variable profit but were predicted to be least impacted by climate change (Harrison et al., 2017). Each of the farm development options had its own strengths and weaknesses, but not many of the options were predicted to always be more profitable than the base farm where the current production system on the case study farm system was modeled.

The social research highlighted further risks and opportunities associated with the farm development options (Table 1). The social license of Intensified options was considered to be more vulnerable than the other options because the increased use of feeding and housing infrastructure with less grazing of pasture could be perceived by the general public as having an animal welfare and/or environmental issues. These options also had potential for increased stress for farming families and farm employees due to the operational precision required to manage larger herds with greater levels of supplementary feeding. The Adapt options were generally considered favorably compared with the Intensified and Simplified options. The key advantages of the Adapt development options were that the farm decision-makers were in a position to take advantage of favorable climatic and market conditions without the level of risk and investment required by the Intensified option; yet, it could generate enough cash flow to maintain the farm business capital and assets for future development or family succession. The Simplified options were identified as a potential risk to regional communities because if there was a major shift to these lower-production systems, there could be a loss of jobs in both the dairy and allied industries and spending in regional economies. Across all the development options, the regional working groups recognized that the dairy industry would require support to adapt to climate change in the form of resources and information for learning and professional development of dairy farm business owners; strong peer networks for social learning; regional workforce strategy to ensure labor availability and capacity development; access to relevant and high-quality agricultural services providers (i.e., consultants, input suppliers, and financial institutions); and community well-being infrastructure such as mental health support services and social networks.

**Table 1.** Opportunities, risks, and tradeoffs from the DBFC development options expressed relative to the Base farm

	Intensify	Adapt	Simplify
Opportunities	• Economies of scale • Can take advantage of favorable conditions • On-farm employment • Feed infrastructure to cope with variable conditions	• Flexibility in directing the business trajectory • Less vulnerable in unfavorable operating conditions • Feasible family business model	• Health and lifestyle advantages • Labor saving by using household members • Stable annual profit
Risks/tradeoffs	• “Lock-in” effects from investing in infrastructure • Psychological stress on staff • Greater variability in profit • Possible environmental issues • Public may perceive an animal welfare issue.	• Moderate capacity to take advantage of favorable conditions • Added complexity combining grazing and mixed ration feeding • Mistakes can be costly in a partial mixed ration system	• Low capacity to take advantage of favorable conditions • Greater reliance on making own decisions • Less capacity to support the regional economy • May limit farming succession

This is an aggregation of findings from the case study regions that emerged from 7 focus groups involving 44 dairy business managers and advisers.

### Benefits and limitations of the transdisciplinary approach

The transdisciplinary approach in the DBFC project brought together multiple academic disciplines with expert knowledge of farmers, advisors, and industry professionals to support experiential learning. The range of research approaches used allowed both technical and nontechnical issues to be explored, with the nontechnical issues identified as significant drivers of change. The process of collaboration in the project team and regional working groups enabled credibility and trust among participants to be established and built, and as a result of this, rich and comprehensive discussions were achieved. This attention to research process can counter maladaptive decisions by providing opportunities for multiple feedback loops and iterations of ideas and proposed actions among participants.

The benefits of the transdisciplinary approach were identified by regional working group members as learning from the diversity of people and perspectives involved in the DBFC project; experiencing how research is done through direct engagement with it; and establishing credibility for the research to influence practice and policy change in dairy industry development. For example, members of the regional reference groups noted the value of the approach as:

*Build[ing] collaborative relationships which helps all parties better understand the research work and the viewpoints of others* (South Australian regional working group members, June 18, 2016).

Another reference group member noted what they had learnt about doing research:

*It’s been great to see how the research has been done, how it has come about, what sort of inputs we (regional working group members) we’ve had into it and then to get out the other end some really good, credible facts, and I’ve found that fascinating.’* (Gippsland regional working group member, April 18, 2016).

And yet another recognized the need to ground research in the realities of dairy farming:

*We’re talking about (dairy) farming systems…obviously you need to have the economics, you need to have the biophysical modeling and the researcher skills…but the farmers have to be there (in the research process) to bring the practical, day-to-day knowledge and challenges. You have to have farmers involved in this kind of project…otherwise you’re not going to come up with any relevant findings that people can take away.* (South Australia regional working group member, June 18, 2016).

Limitations of the approach used in DBFC project include that the focus on engagement with the regional dairy industry may have constrained thinking about adaptation to “dairy production” and not considered the opportunities for more transformational adaptation, for example, consideration of changing production focus to other industries, dairy farming in different locations, or accessing alternative markets. Engaging more broadly with dairy and other agricultural industry stakeholders as a component of the project would help to alleviate this risk of maladaptation.

**A Trip to the Future**

For each of the three case study farms used within the DBFC project, researchers identified locations that were likely to reflect their future climate. The climate projections suggested that in 2040 the climate in Central Gippsland would be similar to the current climate at Tallangatta in north-east Victoria but without the temperature extremes (Figure 2b).

**Figure 7.**
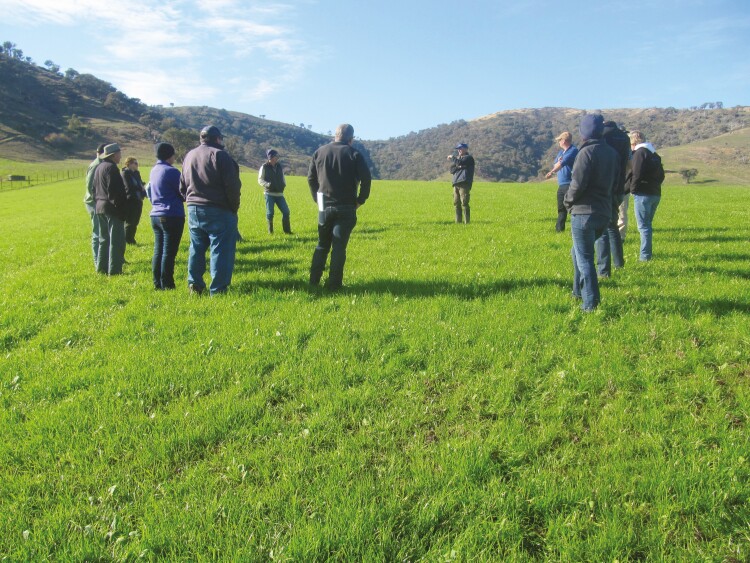
Gippsland regional working group members on a farm tour to north-east Victoria, Australia, to experience dairy production in a region with a similar climate now to that predicted for Gippsland in 2040 (Photo credit: G.H.).

In May 2015, ten members of the regional working group from Gippsland traveled to north-east Victoria to visit dairy farm businesses and meet with a range of farmers and advisors (Figure 7). The purpose of the visits was to see farm systems that were operating in a similar way to the Adapt development option that was modeled for Gippsland. It was an opportunity to explore these systems and further the thinking around possible future pathways for dairy farms in the Gippsland region. Some of the questions explored included:

1. How are these farming systems different from dryland farms in Gippsland?
2. What are the specialist skills required to run these systems?
3. What risk management practices are in place to manage the variable climate?
4. How could these systems translate to the Gippsland dairy industry?

The lessons for the regional working group from this visit included: the adaptive nature of on-farm management; adjusting to seasonal variability and the shortening of the ryegrass growing season; the strong dependence on homegrown fodder including silage reserves and seasonal cropping; and the need for sound pasture management skills as well as looking ahead to assess seasonal risks with an adaptive mindset.

A key benefit for the DBFC project from the visit included the opportunity for researchers and regional working group members to physically visit a different location reflecting a potential future climate. This was an ideal complement to the modeling of future climate scenarios as it engaged different learning styles. The trip also facilitated more farmer-to-farmer learning that made a unique contribution to the project.

## Conclusions

The transdisciplinary research approach used in the DBFC project was an effective way to address the challenge of climate change adaptation in the dairy industry of south-eastern Australia. Climate change impacts are locally specific and multifaceted, so adaptation requires that researchers collaborate with farmers to develop solutions that are grounded in the realities of their industries and communities. The farm development options were a particularly important part of the DBFC project design because they enabled discussion and debate between scientists and industry experts, and thus the integration of different knowledge and insights of the risks and opportunities of adaptation. The transdisciplinary approach utilized in this project is a model that can be modified to suit climate change adaptation research in a broad range of agricultural production systems from smallholder farms in developing countries to intensive dairy production systems. The success of the approach lies in bringing together farmers, advisors, and researchers from different disciplines in an iterative process that develops new and actionable knowledge for climate change adaptation.

## About the Authors

**Brendan R. Cullen** is a senior lecturer in grazing systems in the Faculty of Veterinary & Agricultural Sciences at The University of Melbourne. Over the last 20 yr, he has worked on a series of research projects with the dairy and red meat industries to understand the impacts of projected climate changes on livestock production businesses across Australia. The research aims to identify industry pathways that are adapted to a variable and changing climate. He has completed a Bachelor of Agricultural Science degree and PhD in pasture plant ecophysiology from The University of Melbourne.

**Margaret Ayre** is a senior lecturer in the Faculty of Veterinary and Agricultural Sciences at the University of Melbourne. She has an undergraduate degree in Forest Science (University of Melbourne) and a doctoral degree from the Arts Faculty of the University of Melbourne. She has worked as an environmental policy maker and social scientist in the fields of agricultural development and environmental management for over 20 yr. Her main research interests are in transdisciplinary and cross-cultural knowledge-making, digital agriculture, climate change adaptation, and participatory approaches to supporting resilience and collective action in agriculture and land and water management.

**Nikki Reichelt** currently holds a Research Fellow position with the Rural Innovation Research Group, Faculty of Veterinary and Agricultural Sciences at the University of Melbourne. Over the past 12 yr, she has been involved in rural research at the University in Melbourne after she completed her Masters in Environment at the University of Melbourne. Her research interests include improving agricultural extension and adoption, advancing the thinking in Agricultural Innovation Systems, and supporting climate change adaptation in the livestock industries and the role of rural communities in natural resource management.

**Ruth A. Nettle** leads the Rural Innovation Research Group at the University of Melbourne and is a social scientist focusing on understanding and improving rural innovation processes. Her research examines farm system resilience, technological change, the social organization of farming systems, rural workforce planning and action, and agricultural extension policy and design. She applies engaged research and transdisciplinary research practices to address agricultural and rural community challenges.

**Gillian Hayman** is a consultant working in the fields of agriculture, natural resource management, and community engagement. She has worked in rural communities across south-eastern Australia for the past 25 yr. She is a member of the Victorian government’s Agriculture Climate Change Council and a partner in a dairy farm business in South Gippsland, Victoria. Her interests include sustainable food production systems, adaptive agricultural systems, and thriving rural communities. She holds a Bachelor of Applied Science (Environmental Science) from Charles Sturt University, Graduate Diploma (Land Rehabilitation) from University of Ballarat, and Diploma in Secondary Education (Monash University).

**Daniel P. Armstrong** is the principal consultant at D-ARM Consulting and holds both Bachelor’s and Master’s degrees in Agricultural Science. He has worked as a private consultant since 2010 specializing in providing services in farm systems analysis, economics, and farm business management to the Australian agricultural sector, and particularly to the dairy industry. Previously, he worked for Agriculture Victoria (Kyabram and Ellinbank) for 14 yr on projects relating to dairy farming systems economics and water-use efficiency. He has also been employed by LaTrobe University as a sessional lecturer in Farm Management Economics since 2013. His areas of expertise include economic and risk analysis, farming systems analysis, irrigation, and water policy and extension. He has extensive experience in collaborative farm systems research projects with active involvement in a wide range of multidisciplinary action research teams involving dairy farmers, consultants, and researchers over the last 25 yr.

**Ruth Beilin** is an honorary fellow and professor of Landscape and Environmental Sociology, School of Ecosystem and Forest Sciences, Faculty of Science, University of Melbourne. She has a biophysical and social science background, and her research is in social–ecological systems pertaining to land and water management.

**Matthew T. Harrison** is an associate professor and is Systems Modelling Team Leader at the Tasmanian Institute of Agriculture (TIA) in the University of Tasmania, Australia. Prior to employment at TIA, he worked at INRA in Montpellier, France, Pioneer Hi-Bred in the United States, the University of Queensland in Brisbane, Australia, and at CSIRO in Canberra. He is particularly interested in adaptation of livestock and cropping systems to climate change and extreme climatic events, as well as greenhouse gas mitigation, genotype by environment by management interactions, and big data and machine-learning applications. He works in multidisciplinary teams often in collaboration with end users such as farmers, industry, civil society, and governments at all levels to ensure that his work has economic, environmental, and social impact that is beneficial not just to agricultural stakeholders but also to society at large.
